# Innate and adaptive effector immune drivers of cytomegalovirus disease in lung transplantation: a double-edged sword

**DOI:** 10.3389/frtra.2024.1388393

**Published:** 2024-06-10

**Authors:** Reena Bharti, Daniel R. Calabrese

**Affiliations:** ^1^ Department of Medicine, University of California, San Francisco, San Francisco, CA, United States; ^2^ Department of Medicine, San Francisco Veterans Affairs Medical Center, San Francisco, CA, United States

**Keywords:** lung transplantation, cytomegalovirus, heterologous immunity, allograft injury, effector immune response, NK cells

## Abstract

Up to 90% of the global population has been infected with cytomegalovirus (CMV), a herpesvirus that remains latent for the lifetime of the host and drives immune dysregulation. CMV is a critical risk factor for poor outcomes after solid organ transplant, though lung transplant recipients (LTR) carry the highest risk of CMV infection, and CMV-associated comorbidities compared to recipients of other solid organ transplants. Despite potent antivirals, CMV remains a significant driver of chronic lung allograft dysfunction (CLAD), re-transplantation, and death. Moreover, the extended utilization of CMV antiviral prophylaxis is not without adverse effects, often necessitating treatment discontinuation. Thus, there is a critical need to understand the immune response to CMV after lung transplantation. This review identifies key elements of each arm of the CMV immune response and highlights implications for lung allograft tolerance and injury. Specific attention is paid to cellular subsets of adaptive and innate immune cells that are important in the lung during CMV infection and reactivation. The concept of heterologous immune responses is reviewed in depth, including how they form and how they may drive tissue- and allograft-specific immunity. Other important objectives of this review are to detail the emerging role of NK cells in CMV-related outcomes, in addition to discussing perturbations in CMV immune function stemming from pre-existing lung disease. Finally, this review identifies potential mechanisms whereby CMV-directed treatments may alter the cellular immune response within the allograft.

## Introduction

Cytomegaloviruses (CMVs) are double-stranded DNA viruses that have co-evolved over millions of years with their mammalian hosts (1). CMV is ubiquitous and belongs to the beta subfamily of Herpesviridae (2). It possesses two sets of genetic material, a lipid envelope that encases its icosahedral nucleocapsid, and a proteinaceous tegument that is rich in viral phosphoproteins, including pp65 (3). During acute infection, human CMV (HCMV) interferes with antigen loading through major histocompatibility complex (MHC) downregulation, inhibits T cell receptor co-stimulation, produces viral peptides that imitate inhibitory cytokines which disrupt lymphocyte migration, and downregulates Natural Killer (NK) cell activating ligands while upregulating inhibitors of NK cell function (4). Following acute infection, CMV enters a dormant, or latent, state. While the complete viral genome remains within the host cell during latency, the virus's expression is severely restricted, resulting in very few viral antigens and no viral particles (5). CMV establishes latency in many cell types, though has notable tropisms for myeloid (CD34+) bone marrow progenitor cells, endothelial cells, circulating CD14+ monocytes, and lung and mucosal epithelial cells (6). After solid organ transplantation, reactivation of CMV is provoked by immunosuppression, other infections, or organ injury (7, 8). Reactivation of latent CMV can cause multiorgan disease, and has been associated with reduced graft survival, graft-vs.-host disease, post-transplant lymphoproliferative disorders, and increased mortality (9).

CMV is a leading cause of morbidity after lung transplant (LT) (10, 11), where up to 70% of lung transplant recipients experience CMV reactivation or primary infection within the first post-transplant year (12–16). CMV can lead to pneumonia, pneumonitis, and direct extra-pulmonary organ involvement (17). In addition, CMV has been associated with risk for several chronic lung transplant clinical syndromes including antibody-mediated rejection (AMR) and chronic lung allograft dysfunction (CLAD), the syndrome of chronic rejection (18–21). Lung transplant CMV complications increase care costs by 50% when compared to recipients who do not develop CMV disease after lung transplantation (22). Moreover, the extended utilization of CMV antiviral prophylaxis is not without drawbacks, often necessitating treatment discontinuation due to intolerance, drug interactions, leukopenia, or broader bone marrow suppression. There is emerging evidence that these poor CMV-related outcomes in lung transplant recipients are driven by a dysregulated immune response (23).

### CMV exerts tissue-specific effects which impact tissue-level immune responses

CMV infection and its effects on allograft function are confounded by differences in cellular receptors and viral gene expression across tissues (8). The diversity of CMV strains and their adaptation to different tissue types is a complex phenomenon that likely involves a combination of factors, including genetic variation among CMV strains and viral evolution within the host (24, 25). Notably, different strains of the virus may have specific tropisms for different tissues. For example, certain strains of CMV may preferentially infect the lungs, while others may be more adept at infecting the liver or brain. This strain-specific tropism can contribute to the observed differences in CMV infection across different tissue types (26). In a study investigating differences in CMV genome by tissue, there were eighty viral genes with differential tissue expression levels (27). Because of its tissue specificity, CMV may activate different transcriptional processes in different cell types. Post-transcriptional events, such as mRNA stability, may also differ by target tissue or be influenced by local factors such as cytokines or microbes (28). Additional factors that influence the impact of viral infection include variations in immunosuppressive regimens and genetic vulnerability. It has also been shown that CMV microRNAs (miRNAs), short noncoding RNA species, differ by tissue type and may allow viral gene expression to be tissue specific (28–30). Certain miRNAs were also identified that reduce the cellular stress response, decrease cytokine release, and that downregulate host pattern recognition receptors (31–33). Finally, different tissues may exhibit variations in immune surveillance and response, which can influence the course of CMV infection and the selection pressures acting on the virus within each tissue microenvironment.

### CMV is associated with a high burden of disease in lung transplant recipients

The incidence of CMV disease after lung transplantation ranges from 30% to 86%, with a CMV-attributable death rate of 2%–12% (34). This increased burden in LTRs may be a consequence of the lung as a reservoir for CMV latency (35) where higher viral loads are retained in the extensive pulmonary lymphatic system as compared to other solid organs transplants (33). It follows that CMV severity varies based on donor and recipient CMV serostatus (36). Donor seropositive and recipient seronegative (D+/R−) solid organ transplant recipients have the highest risk of CMV disease (37), while D−/R− recipients have the lowest risk for CMV disease (38). The other donor-recipient CMV pairings (D+/R+ and D−/R+) confer intermediate risk. Another driver of lung transplant CMV stems from the higher doses of induction and maintenance immunosuppression compared to other solid organ transplants (37). Antilymphocyte induction therapy agents, such as alemtuzumab or anti-thymocyte globulin have been associated with a nearly 6-fold increased incidence of early CMV reactivation after transplantation (39–41). Comparatively, non-depleting induction regimens, such as those that use basiliximab, may confer a reduced risk for CMV reactivation (42). Other factors prevalent in the lung transplant population can amplify the impact of CMV and lower the threshold for CMV reactivation. These include telomere biology disease, inherent disorders of immune function, and acute rejection or acute injury syndromes (43, 44).

### Impact of immunosuppressive drugs on CMV

Immunosuppressive agents, vital for preventing organ rejection in transplant recipients, impact the risk and management of CMV infection. For example, Calcineurin Inhibitors (Cyclosporine and Tacrolimus) are cornerstone drugs in the transplant immunosuppression regimen and have been shown to predispose patients to viral infections, including CMV, by broadly suppressing the T cell immune response (45). Mycophenolate Mofetil (MMF) and Azathioprine inhibit the proliferation of T and B cells, reducing the immune response to both the transplanted organ and infectious agents like CMV. Notably, the degree of immunosuppression correlates with the risk of CMV infection (46). Mammalian Target of Rapamycin (mTOR) Inhibitors (Sirolimus and Everolimus) have impact on CMV infection (47). Alemtuzumab is a potent immunosuppressive agent but significantly increases the risk of infections, including CMV, by depleting lymphocytes, a critical component of the antiviral immune response (48).

Mean residual expression (MRE) could be a valuable tool in identifying lung allograft recipients who are at an increased risk of infection, by identifying the functional effects across immunosuppressive agents on the recipient immune response (49). This could potentially lead to more personalized immunosuppression regimens that balance the risk of rejection with the risk of infection, including CMV reactivation (48).

### The early anti-CMV immune response is marked by monocyte and macrophage activity and immune evasion

CMV, along with other human herpesviruses, primarily infect fibroblasts, endothelial cells, epithelial cells, monocytes, and macrophages. It gains entry through the cell membrane via glycoprotein B (gB), which is a viral enveloped fusion glycoprotein; though, whether entry is mediated through gB-receptor dependent or independent processes is unknown (50). Potentially through caspase 2 activation, there is a subsequent release of inflammatory cytokines from these infected cells including TNF-α, IL-6, in addition to a robust type I interferon (IFN) response (51).

The heterogeneous macrophage (MΦ) population performs infection surveillance and maintains tissue homeostasis. MΦ function, and even reprogramming, occur in response to circulating mediators (52). Macrophages have been well described as hewing to two polarized differentiation pathways, M1 and M2, which have been named after CD4+ T helper (Th) cell subtypes (53). M1 (proinflammatory) MΦ's secretes IL-1β, IL-6, IL-12, and TNF-α, unlike M2 MΦ (anti-inflammatory) (54), which primarily secrete IL-10 and have other phenotypic and functional differences (55). The lungs have functionally distinct subsets of MΦ, notably, alveolar MΦ and interstitial MΦ that have been shown to be immunosuppressive with a poor ability to present antigens (56). Nonetheless, CMV infection triggers the production of pattern recognition receptor ligands. These ligands bind to TLR4 and TLR5 on macrophages and lead to intracellular MyD88 signalling and gene transcription of the proinflammatory cytokines TNF-α, IL-6, and IL-8 (57).

While the early inflammatory cascade of CMV infection is mediated by macrophages, HCMV has been shown to evade immune recognition by dampening the macrophage-specific response. MΦ's infected with HCMV downregulate MHC class I and class II molecules as well as chemokine receptors (56, 58). Concordantly, HCMV-infected monocytes differentiate away from an M1 phenotype and toward a mixed M1-M2 MΦ phenotype, which ensures successful viral survival and persistence (58). Persistent macrophage infection can overwhelm the conventional clearing mechanisms of the immune system (59). Further immune evasion occurs through reduction in the production of ligands to activating receptors on effector immune cells (60). The CMV protein UL141 directly binds to some of these ligands in macrophages and in other cell types (61). It has also been observed that noninfected bystander MΦ's are also impacted during CMV infection. This implies that tissue signals and soluble mediators may indirectly play a role in dampening the CMV-specific immune response (62).

In lung transplantation, CMV infection may drive a dysregulated macrophage response which contrasts to conventional macrophage responses to other viral infections (63, 64). This largely stems from the co-evolution of macrophages with CMV, as they are a primary host cell for CMV infection (65). Activation of TLR4, an important receptor in the early CMV immune response, has been closely linked to acute lung allograft rejection (66, 67). Bronchoalveolar lavage samples from lung transplant recipients with CMV pneumonia demonstrated increased gene expression of IL-1, IL-6 and serine esterase B, with concordant findings during acute rejection (68). Significant increases in the concentrations of cytokines, such as CXCL-10, CXCL-16, CCL-18, and CCL-20, in bronchoalveolar lavage have also been observed during HCMV replication in lung transplant recipients. The effector CD8+ and CD4+ T lymphocytes are selectively attracted by CXCL-10 and CXCL-16 (69). Antigen-presenting cells generate CCL-18, which acts as a monocyte activator and chemoattractant for B-cells, immature dendritic cells (DCs), and naïve and regulatory T cells. Pulmonary epithelial cells and monocytes secrete CCL-20, which binds to CCR6, a specific receptor on T cells and immature dendritic cells (DCs). CCR6 is prominently expressed on Th17 CD4 cells, making them particularly susceptible to the chemotactic effects of CCL-20. In addition, Th17 CD4 cells have been implicated in acute rejection (70, 71). IL-17 promotes neutrophil chemotaxis via the induction of IL-8. This leads to an increased presence of neutrophils and lymphocytes within the lung tissue, contributing to an inflammatory response characteristic of acute rejection (70). Thus, CMV-associated activation of macrophages may drive maladaptive T cell responses in the lung allograft.

## Lymphocytes in CMV, in the lung and in transplantation

### NK cells play an important role in mitigating CMV

Natural killer cells are lymphocytes and constituents of the innate immune system. Their primary role is to identify missing, transformed, or damaged self (72, 73). As opposed to T and B cells, where specificity for antigens occurs through genetic rearrangement, NK cell activity is dependent upon the integration of activating and inhibiting signals from somatically encoded receptors (74). There has been an increasing awareness that NK cells play a central role in many lung transplant conditions (75), and nowhere is their function in the lung more evident than during CMV infection.

NK cells are key mediators of CMV infection, where they eliminate CMV-infected cells through antibody-dependent cell-mediated cytotoxicity and through the modulation of T and B cells via the secretion of IFNγ (76). NK cells recognize CMV via the C-type lectin-like receptor CD94/NKG2C in humans and the Ly49H receptor in mice (77, 78). In humans, T cells also express the NKG2C receptor. Immunoreceptor tyrosine-based activation motifs (ITAMs) are located on the DAP12 signalling adaptor, which covalently binds NKG2C with the CD94 glycoprotein. This complex functions as a ligand-recognition mechanism for the invariant HLA-E protein (79). It is still unclear which CMV-related antigen is presented on HLA-E. While it is possible that HLA-E directly presents a dominant CMV peptide epitope, none has been identified to date (80). It is more likely that NKG2C+ NK cells may recognize changes in HLA-E stability and HLA-E-associated molecules that are presented during CMV infection (81). In parallel in the mouse, the NKR Ly49H receptor enables direct identification and killing of mouse CMV (mCMV) infected cells via recognition of the m157 protein (82–84).

In humans and mice, NKG2C or Ly49H activation leads to potent NK cell effector functions and results in memory-like capabilities. In a mouse model of CMV infection, mortality was improved in animals that received NK cells from mice previously infected with CMV as compared to mice with NK cells transferred from unexposed mice or those without NK cell transfers (85). In both human and mouse, these CMV-specific NK cells undergo avidity selection for NK cell clones that have the highest virus-specific receptor, expand, and persist as long-lived memory cells (83, 86). Recently, it has been shown that these long-lived CMV-specific NK cells localize to and persist in the lung and salivary gland tissue (87).

### The NK cell response to CMV in lung transplantation

After transplantation, HCMV induces potent NKG2C+ NK cells (88–90). In kidney transplant recipients, CMV seropositive recipients demonstrated increased frequencies of baseline memory-like NK cells that expressed CD57 (88) and lacked FcεRIγ. Longitudinally, this population was dynamic and decreased in the circulation after transplant and after CMV reactivation. In this same group, a pre-memory NK cell population defined by CD57 and FcεRIγ^low^ expression increased during CMV reactivation. This population had increased proliferative and cytotoxic capabilities. These findings demonstrate the interplay between transplantation and CMV in influencing CMV-specific cell populations and demonstrates that even in the presence of immunosuppression, transplant recipients can generate new innate effector memory (91).

There is evidence that NKG2C+ NK cells are important in constraining CMV infection in lung transplantation. A large percentage of the human population (∼70%) lacks the NKG2C encoding gene, *KLRC2* (92). In a study of 98 lung transplant recipients, participants with intact *KLRC2* alleles were less likely to experience CMV DNAemia and CMV-associated disease compared to participants harbouring the null allele (93). In a study of 130 lung transplant recipients, bronchoalveolar lavage NKG2C+ NK cells were increased in CMV seropositive individuals at baseline and expanded preceding CMV DNAemia (94). These studies suggests that NKG2C+ NK cells play a potentially crucial role in the CMV immune response following lung transplant.

### The CD8 T cell response to CMV

*In vivo* data suggest CMV-specific CD8+ T lymphocytes are the primary population responsible for limiting viral cell replication (95). In genetically resistant mouse inbred strains (96), NK cells mediate early protection, whereas CD8 T cells acquire long-lasting protective memory and serve as primary antiviral effectors in susceptible strains (96, 97). Notably, studies using CMV tetramers, demonstrate that CMV-specific T cells are most abundant in blood (98) and lungs. Infection with CMV also results in the production of distinct CD8+ T-cell phenotypic population (99). Most CMV-specific CD8 T cells are effector memory cells (T_EM_), noted by the absence of CD62L and CCR7, 2 lymph node homing markers (100). CMV-specific CD8 T_EM_ notably downregulate co-receptors (CD27 and CD28) and have markers of maturation (CD57, KLRG1) and increased effector functions (101) Memory CD8+ T cell responses to CMV have received a great deal of attention, owing to their atypical expansion, known as memory inflation (102), which occurs to a lesser extent in CMV-specific CD4+ T cell populations (103). Although these populations are thought to be associated with lifelong CMV infection, the mechanisms underlying these expansions and how they are maintained remain unknown.

In human lung transplant, CMV-specific CD8 T cells vary less in the blood than in the lungs where they have been reported to be increased preceding CMV replication (104). During CMV infection, studies have reported a profound influx of CD8 T cells into the lung allograft (105). These lung allograft CD8 T cells express an effector memory phenotype, demonstrate CMV-specificity, and undergo a contraction followed by long-term persistence after CMV infection. In a study of 41 lung transplant recipients, CMV DNAemia was associated with the expansion of 3 distinct populations of terminally differentiated effector memory (TEMRA). CD8 T cells in the circulation marked by differences in CD57 and KLRG1 expression. CD57 + KLRG1+ CD8 TEMRA cells were associated with CMV clearance and reduced risk for chronic lung allograft dysfunction or death (106, 107). Together, these data suggest that a robust CD8 T cell memory response can contribute to improved CMV control and better long-term outcomes after lung transplantation.

### The γδ T cell response to CMV

Among lung transplant recipients with high-risk CMV mismatches (D+/R−), effector γδ T cells increased longitudinally after transplant, exhibit increased frequencies of the NK cell NKG2C receptor, and demonstrate increased T cell receptor diversity (108).

T cells with the γδ T cell receptor contribute to anti-cancer and anti-infection immune responses (109, 110). γδ T cells are different from αβ T cells in several ways, most notably in antigen detection and effector destiny development, even though they perform similar vital tasks (111). In contrast to αβ T cells, major histocompatibility complex (MHC) does not limit the activity of γδ T cells (112). Also, the variety of ligands recognized by the γδ TCR is remarkable and includes MHC-related proteins in addition to low molecular weight non-peptide ligands (113). Following CMV reactivation in transplant patients, γδ T cells increase and persist in the circulation (114). The observation that γδ T cells are protective from CMV-associated mortality in mice deficient in αβ T cells suggests that γδ T cells play a protective role in mitigating CMV disease (115). In lung transplant, it has been observed that recipients with high-risk CMV mismatches (D+/R−) have a robust effector γδ T cell response after transplant, increased expression of the NK cell NKG2C receptor, and increased T cell receptor diversity (108).

### Other lymphocyte response to CMV

In the immunological response against CMV during lung transplantation, CD4+ T cells and B cells are essential components. Adoptive transfer of CMV-specific CD4+ T cells into sub-lethally irradiated mouse hosts show a protective effect. These cells are more effective when administered in conjunction with CD8+ T cells specific to the virus (116). A delayed emergence of CMV-specific CD4+ T cells is linked to longer viremia and more severe clinical illness in immunosuppressed solid organ recipients (117). In trials of cytotoxic T cell therapies in humans, CMV-specific CD4+ T cells are necessary for the persistence of CMV-specific CD8+ T cells (118). Further, CD4+ T cells may facilitate the entry of naïve CD8+ T cells and B cells to draining lymph nodes and serve to entice innate or antigen-specific effectors to viral replication sites through the secretion of local chemokines and the synthesis of IFN-γ. Antigen binding on CD4+ T cells triggers CD40L expression, which activates CD40 on B cells and promotes B cell proliferation and differentiation, initially in extra-follicular foci and then in lymph node germinal centers, producing memory B cells and plasma cells that generate anti-CMV antibodies (119). CD4+ T cells promote memory CD8+ T cell development through a variety of mechanisms, including downregulating the expression of TNF-related apoptosis-inducing ligand (TRAIL), producing cytokines like IL-2, or directly ligating CD40 on naïve CD8+ T cells (120, 121). The critical role that CD4+ T cells play in antiviral immunity is highlighted by the existence of viral genes that down-regulate MHC class II molecules and the expression of viral IL-10, both of which restrict antigen presentation to CD4+ T cells (104, 118).

In the spleen, B cells initiate the early interferon response to CMV in a lymphotoxin receptor-dependent manner (121). Moreover, they play a role in mitigating CMV infection via the production of anti-CMV antibodies. Though, the observation that B cells are decreased in lung transplant recipients with CMV and CLAD highlights the uncertainty in their role in the lung (122, 123). Additional investigation is needed to fully understand how B cells, specifically within the allograft, may contribute to CMV pathology.

## The role of heterologous immune responses in promoting tissue- and allograft-specific immunity

Heterologous immunity as a phenomenon was recognized early in the infection literature but has profound implications for autoimmunity and allorecognition. The concept of heterologous immunity centres on the observation that previously encountered antigens can alter immunity to new or novel antigens (124). This heterologous immune response can be helpful or harmful to the host and depends largely on cellular and tissue contexts. Thus, the chronic immunological responses required to control CMV infection may cause graft harm by direct antibody-dependent cell-mediated cytotoxicity, the formation of heterologous alloimmune responses, and proinflammatory cytokine production (125).

### CD8 T cells and heterologous immunity

The observation that there are more recognizable antigens than unique T cell receptors (TCRs) supports evidence of T-cell cross-reactivity (126). The mechanism whereby this is achieved remains to be fully elucidated but may result from the ability of the same TCR to recognize 2 different peptide-MHC complexes. Consequently, infection with CMV, in addition to a variety of other viruses, has been observed in C57BL/6 mice to promote CD8 T cell specificity to MHC I antigens in addition to viral antigens (127). In an allogeneic model of rat lung transplantation, infection with parainfluenza 1 virus broke tolerance and induced obliterative airway disease (128). Such findings suggest a heterologous component to lung transplant rejection following viral infection.

### NK cells and trained immunity

While circulating and allograft NKG2C+ NK cells in lung transplant recipients expand with CMV reactivation, there is evidence that this cell population may have a dual role in allograft outcomes (9, 129). Among 130 lung transplant recipients, higher than the median NKG2C+ NK cells in the BAL conferred more than a 4-fold increased risk for CLAD or death (94). This suggests that NK cells may be recognizing low-level chronic CMV infection in the lung and potentiating chronic injury to the airways (18). In this same study, NKG2C+ NK cells were shown to have increased CD16, an activating-only Fc receptor. Indeed, NKG2C+ NK cells retain potent effector functions including the ability to participate in antibody-dependent cell-mediated cytotoxicity (ADCC) (130). In a longitudinal study of bronchoalveolar lavage NK cells after lung transplantation, CD16+ NK cells were associated with acute lung allograft dysfunction and antibody mediated rejection (AMR) (131). Further, increased CD16+ NK cells among recipients with AMR conferred an increased risk for chronic lung allograft dysfunction.

There is emerging evidence that CMV may influence the ability of NK cells to participate in antibody mediated rejection after lung transplantation. UL40 peptides, produced by CMV, have been shown to alter expression of HLA-E which binds NK cell receptors. Through a variety of potential mechanisms, UL40 may alter the NK cell response to CMV-infected cells, and polymorphic UL40 peptides induce variable NKG2C+ NK cell effector functions (132). Indeed, in a study of 150 lung transplant recipients, certain UL40 peptide variants were associated with AMR and induced proliferation of CD16 + NKG2C+ NK cells (92). Interestingly, in a cohort of 82 lung transplant recipients with at least 1 episode of CMV reactivation, one UL40 peptide variant was associated with CLAD and impeded immature NK cell proliferation. These data reveal a direct interaction between CMV peptides and NK cell functional phenotypes in mediating AMR and CLAD. While not meeting the true definition of heterologous immunity, these data illustrate how CMV infection may lead to off-target effects in lung allografts.

### There is an interplay between CMV and allograft function

Lung allograft rejection is typically classified into acute cellular rejection and antibody-mediated rejection. Acute cellular rejection is characterized by perivascular and interstitial mononuclear cell infiltration, predominantly by lymphocytes. In contrast, antibody-mediated rejection involves capillary inflammation with neutrophils and evidence of antibody deposition, often detected through immunofluorescence. The diagnosis of rejection is based on histological examination of lung biopsy specimens. As discussed, CMV infection may cause direct and indirect allograft injury through a range of mechanisms (8). Consequently, acute allograft rejection also has implications for CMV pathogenesis directly. The production of tumour necrosis factor-alpha (TNF-α) in lung transplant recipients as been shown to promote CMV replication and is a critical driver for CMV reactivation from latency. Remarkably, the CMV viral protein pUL138 also amplifies the reaction to TNF-α by upregulating the receptor's cell surface expression (24).

Consequently, the pathogenesis of vascular injury observed in acute and chronic rejection is likely influenced by CMV infection of smooth muscle cells and vascular endothelium (133). The hallmark of CMV infection in lung transplant shows characteristic cytopathic effects, such as enlarged cells with intranuclear inclusions (“owl's eye” appearance) and intracytoplasmic inclusions. Real-time polymerase chain reaction (PCR) for CMV DNA quantification in blood or tissue samples is another crucial diagnostic tool, offering both high sensitivity and specificity for detecting viral replication. Though, our understanding of the pathology of heterologous CMV immunity specifically during lung transplant rejection is limited. In the future, tissue transcriptional analyses may yield insights into the CMV-specific T cell receptor repertoire or overall CMV immune response.

CMV causes an increase in the number of inflammatory cells in the graft by upregulating endothelial adhesion molecules like VCAM, ICAM, LFA-1, and VLA-4. A further mechanism of injury is molecular mimicry through induction of a glycoprotein homologous to MHC class I antigens. There is evidence of sequence homology and immunologic cross-reactivity between CMV immediate early antigens and the HLA-DR β chain (40). According to the “missing self-hypothesis,” loss of MHC class I may activate natural killer cell recognition and killing. As a result, HCMV promotes the expression of an HLA-E inhibitory receptor as well as several gene products that upregulate natural killer inhibitory receptors and disable natural killer activating receptors (59, 103).

A notable finding following CMV pulmonary infection is the development of numerous CMV plaques (134). These plaques have been shown to draw in inflammatory cells to the lung parenchyma. In a mouse model of bone marrow transplantation, CD3ε+ T cells were seen three weeks after viral infection co-localized to CMV-infected cells in the lung (135). The tertiary lymphatics have been shown to be central to tolerance in mouse allogeneic lung transplant (136). Such a CMV-related phenomenon could prime the tissue resident immune populations away from tolerance. Thus, the interplay between CMV and the lung allograft can significantly impact long-term outcomes.

Additionally, there is evidence that perturbations from underlying lung disease may alter the immune response to CMV. Mechanistically, short telomeres trigger ATM-dependent DNA damage repair pathways, which activate p53 signalling. In lung transplant recipients, this has shown to result in the cessation of proliferation and the release of pro-inflammatory cytokines (137). CMV exposure in lung transplant recipients with short telomeres had skewed distributions of conventional CD4 T cells and CD8 T_EMRA_s. Short recipient telomere lengths following transplantation have been sporadically associated with leukopenia, risk of cytomegalovirus (CMV) infection, reduced acute cellular rejection, and worsened chronic lung allograft dysfunction (CLAD)-free survival (138). A study of CD8 T cells in lung transplant recipients with short telomeres and idiopathic pulmonary fibrosis found several CD8 T cell impairments including reduced proliferation and effector functions (23). These data show how the CMV response after transplant may be influenced by systemic drivers of lung disease.

### The impact of CMV-specific therapies on cellular immune populations

There are a host of available agents to manage CMV disease in the lung transplant population; though, CMV eradication is not possible currently. Thus, common CMV management strategies often focus on prophylaxis agents in high-risk lung transplant recipients to reduce the potential for reactivation (139). In addition, there are several strategies for the treatment of CMV disease or CMV DNAemia. Currently, most monitoring protocols involve BAL and plasma surveillance of CMV replication by PCR (140). Some centers have instituted CMV immune assays like quantiferon-CMV or commercial CMV ELISPOT assays designed to gauge cellular immunity (141).

The mainstays of CMV prophylaxis and treatment are antiviral agents, which include ganciclovir, valganciclovir, and the newer agents marabavir and letermovir (142, 143). Foscarnet has been used in the past but has fallen out of favor given its poor safety characteristics (143, 144). Valgancyclovir is the oral prodrug of ganciclovir which works through inhibition of viral DNA synthesis. Valganciclovir has been shown to influence NK cell frequencies and function. In a study of human herpesvirus-8, valganciclovir-treated participants had reduced NK cells in circulation and more pronounced KLRG1 expression (145). In a randomized study of valganciclovir compared to placebo in HIV+ patients, the valganciclovir group had reduced CD8 T cell activation relative to the placebo group (146). Among kidney transplant participants, lymphocyte proliferation and activated T cell counts were reduced with valganciclovir treatment (146, 147). Despite these changes, there is evidence that valganciclovir may preserve the CMV-specific T cell responses after lung transplantation (148).

In addition to antiviral agents, CMV hyperimmunoglobulin (CMVIg) has been used for several decades for prophylaxis against CMV reactivation in high-risk donor-recipient pairings (149). Proposed mechanisms for CMVIg efficacy include reduction of cytokine production, as observed in mixed lymphocyte reactions and anti-CD3 blastogenesis experiments, the reduction of ADCC, induction of CD8 T cell and NK cell apoptosis, and reduction in T-cell proliferation (150). Given these changes, one outstanding question is whether CMVIg may modulate graft outcomes. There is some indication that CMVIg may reduce acute rejection rates following heart or lung transplantation (151); though, the evidence is not conclusive and has not been verified for other solid organ transplant groups (152).

In summary, anti-CMV strategies influence immune populations known to be important in the CMV inflammatory response though, more study is needed to understand whether these occur independent from reduced CMV activity or directly influence allograft outcomes. Recently, it has been theorized that CMV-specific effector cells may be harnessed to treat refractory disease (115). It was demonstrated that expansion and infusion of autologous CMV-specific cytotoxic lymphocytes in a lung transplant recipient with ganciclovir-resistant CMV disease was safe (115). In a prospective trial of a similar strategy in solid organ transplant recipients, *in vitro* expanded autologous CMV-specific T cells were associated with an 84% response rate including blunting of CMV DNAemia (153). Several recipients showed long-term persistence of anti-CMV cellular immunity following this novel treatment. More work is needed to establish the safety and efficacy of this intriguing approach. Finally, the field may see the arrival of an effective CMV vaccine; though, efficacy in transplant populations and the long-term cellular implications for graft function will be important to understand (154).

## Summary

**Figure 1:**
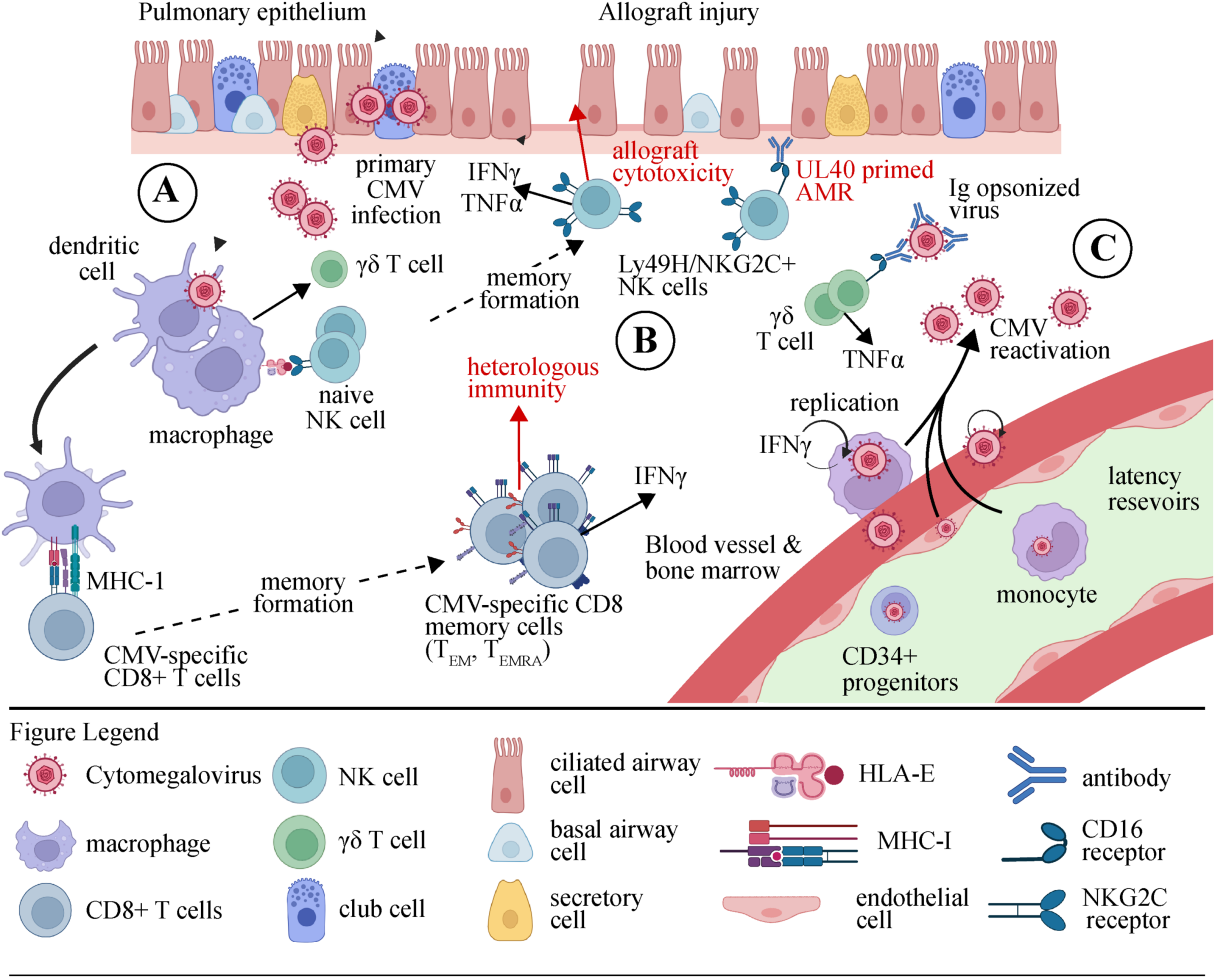
Illustration depicting the cellular response to CMV in the lung allograft. (**A**) During the initial stages of primary CMV infection, phagocytes, and dendritic cells (DCs) are stimulated by viral products through Toll-like receptors (TLRs) and nucleic acid sensors. As a result, they release pro-inflammatory cytokines (IFNαβ, IL-12, and IL-18) and present antigens that trigger the activation of natural killer (NK) cells and γδ T cells. (**B**) The activation of dendritic cells (DCs) results in their maturation and subsequent migration to lymph nodes. The process of presenting viral peptides to inactive CD8+ αβ T cells leads to their differentiation into either effector memory (TEM) or terminally differentiated effector memory (TEMRA) cells, as well as their expansion and acquisition of effector capabilities. Activated natural killer (NK) cells and αβ and γδ T cells can destroy and remove cells infected with cytomegalovirus (CMV) or regulate the replication of the virus by releasing anti-viral cytokines such as IFNγ and TNFα. Allograft injury may occur through this process. (**C**) Although an immune response is mounted, CMV continues to survive within its host. During viral reactivation, immune cells activated by CMV promptly respond to the presence of virions by recognizing m157/HLA-E, stress molecules, or viral peptides. Furthermore, the production of IFNγ by γδ T cells and NK cells stimulated by CMV can be triggered by the interaction between CD16 and Ig-opsonized viruses. CMV, cytomegalovirus; IFN, interferon; Ig, immunoglobulin; NK, natural killer cell; T_EM_, effector memory T cell; T_EMRA_, CD45RA^+^ effector memory T cell.

Figure 1 depicts the impacts of CMV and CMV-specific effector immune cells on the lung allograft. Solid organ transplant recipients face a substantial clinical burden from CMV infection. Though, the rate of significant CMV disease is greater in lung transplant recipients than in recipients of all other solid organs owing to a variety of factors including high viral burden and need for more potent immunosuppression. Despite effective therapies, CMV remains a strong risk factor for CLAD following lung transplantation. While CMV can cause direct lung allograft damage, there is emerging evidence that poor outcomes are driven by effector immune system dysregulation. NK cells, CD8 T cells, and γδ T cells play important roles in mitigating CMV infection. However, after lung transplantation, heterologous immunity and off-target effects in these populations may predominate. Finally, several agents are used to prevent and treat CMV, and each may modulate the CMV immune response.
